# The validity of computerized Montreal cognitive assessment among aging people living with HIV: A pilot study

**DOI:** 10.1186/s12883-025-04425-9

**Published:** 2025-10-01

**Authors:** Akarin Hiransuthikul, Thanapoom Taweephol, Netchanok Timachai, Saowaluk Suksawek, Chuleeporn Wongvoranet, Tanakorn Apornpong, Kittithatch Booncharoen, Solaphat Hemrungrojn, Anchalee Avihingsanon

**Affiliations:** 1 Department of Preventive and Social Medicine, Faculty of Medicine, Chulalongkorn University, Bangkok, Thailand; 2 HIV-NAT, Thai Red Cross AIDS and Infectious Diseases Research Centre, Bangkok, Thailand; 3 Neurological Institute, University Hospitals Cleveland Medical Center, Case Western Reserve University, Cleveland, OH USA; 4 Department of Microbiology, Faculty of Medicine, Chulalongkorn University, Bangkok, Thailand; 5 Neurocognitive Unit, Division of Neurology, Department of Medicine, Faculty of Medicine, Chulalongkorn University, Bangkok, Thailand; 6 Department of Psychiatry, Faculty of Medicine, Chulalongkorn University, Bangkok, Thailand; 7 Center of Excellence in Tuberculosis, Department of Medicine, Faculty of Medicine, Chulalongkorn University, Bangkok, Thailand

**Keywords:** Aging, Cognition, Computerized tool, HIV, Montreal cognitive assessment

## Abstract

**Background:**

As the population of aging people living with HIV (PWH) increases, many have faced neurocognitive problems. Cognitive assessment plays a crucial role as the initial step in cognitive care of this specific population. We aimed to determine the validity between a traditional paper-based and tablet-based cognitive assessment tool among aging Thai PWH.

**Methods:**

PWH aged ≥ 50 years underwent cognitive assessment using the Thai-validated Montreal Cognitive Assessment (MoCA). Participants were randomly assigned to receive either the paper-based MoCA or the tablet-based MoCA (eMoCA) first. Two weeks later, participants returned to complete the alternate version of the MoCA. Pearson correlation was used to determine the strength of the relationship between the paper-based MoCA and the eMoCA scores. Concordance correlation coefficients (CCC) were calculated, and a Bland-Altman plot was employed to determine the level of agreement between the two testing methods. Additionally, MoCA scores were compared between individuals with and without prior touchscreen tablet experience.

**Results:**

Among 46 participants included in the analysis, 12 (26.1%) had experience using a touchscreen tablet. The score discrepancy between the two MoCA versions ranged from − 8 to 6, with a mean (SD) difference of -1.33 (3.22). The Pearson correlation coefficient between the paper-based MoCA and the eMoCA was *r* = 0.54 (*p* = 0.001), with a concordance correlation coefficient of 0.47. The Bland-Altman plot showed 95% limits of agreement between − 7.63 and 4.98. Among participants with prior touchscreen tablet experience, scores between the paper-based MoCA and the eMoCA were comparable. However, those without prior touchscreen experience had significantly lower scores on the eMoCA compared to the paper-based MoCA (mean difference − 1.56, 95% CI -2.72 to -0.40).

**Conclusions:**

The eMoCA demonstrated moderate correlation with the paper-based MoCA, with prior touchscreen tablet experience significantly affecting the validity of the MoCA scores between the two versions. Clinicians should consider individuals’ level of touchscreen experience before selecting the administration modality.

**Supplementary Information:**

The online version contains supplementary material available at 10.1186/s12883-025-04425-9.

## Background

Up to 50% of people living with HIV (PWH) may demonstrate cognitive function that is slightly below the normative mean at some point in their lives [1]. PWH are more vulnerable to such cognitive change due to the combined effects of HIV and aging on neural mechanisms [2]. As aging is the most important risk factor for cognitive decline [3–5], and with PWH living longer due to improved accessibility of antiretroviral therapy, addressing cognitive health in this population is becoming increasingly imperative.

Early detection and intervention are crucial to reduce the risk of developing dementia, including among PWH. Cognitive assessment plays a vital role in the clinical care of individuals suspected of cognitive impairment in PWH. The Montreal Cognitive Assessment (MoCA), developed by Nasreddine and his team in 2005, has proven to be an effective and practical screening tool for mild cognitive impairment (MCI) and mild dementia due to Alzheimer’s disease with good construct validity [6, 7]. The MoCA is a 30-point cognitive screening test comprising various cognitive domains, including visuospatial/executive function (clock-drawing task, three-dimensional cube copy, and Trail Making B task), naming (three-item confrontation naming task with low-familiarity animals), attention (digits forward and backward, target detection using tapping, and serial subtraction task), language (repetition of two syntactically complex sentences and phonemic fluency task), abstraction (two-item verbal abstraction task), delayed recall (short-term memory recall task), and orientation (time and place) assessment that can be completed approximately in 10 min. The MoCA has demonstrated high sensitivity (90%) and specificity (87%) for detecting MCI. The Thai-validated version of the MoCA adjusted the cutoff score from 26 to 25 and added an additional point to participants with ≤ 6 years of education instead of ≤ 12, as in the original MoCA [8]. 

Although the MoCA was not originally validated for PWH, it is considered a promising tool for clinical practice due to its ease of use, accessibility, and coverage of multiple cognitive domains, including subcortical-related domains commonly affected in cognitive impairment in PWH. MoCA has generally shown moderate accuracy in identifying HIV-associated neurocognitive disorders (HAND) in PWH [9]. Data on MoCA scores comparing people without HIV (PWoH) and PWH have also been inconsistent. One study including participants aged 20 to 70 years (mean approximately 45) found that PWH had significantly lower MoCA scores than PWoH.⁹ In contrast, another study conducted exclusively among Thai individuals aged 50 years or older found no significant difference in MoCA scores between the two groups [10]. 

As technology advances, electronic assessments offer several potential benefits, including improved standardization and greater convenience for cognitive screening [11]. These advantages could help address the ongoing issue of low uptake of cognitive assessments in real-world, high-demand clinical care settings, such as HIV clinics. An electronic version of the MoCA (eMoCA) has been developed, utilizing the same assessments but administered on a tablet computer [11, 12]. However, the method of administration may influence test performance, particularly in older adults who may have limited experience with stylus pens and tablet computers, especially in low- and middle-income countries (LMICs), where the ability to use these devices could be constrained by affordability and lower digital literacy, compared to high-income countries (HICs). If the eMoCA proves to be comparable to the traditional version, it could simplify electronic data collection, enhance reliability, and improve clinical efficiency by automating scoring processes. The Thai-validated MoCA has also been adapted into a tablet-based version. Therefore, the opportunity to integrate the eMoCA into the model of care for aging PWH presents a promising approach for cognitive impairment detection and enhancing data acquisition in the setting of LMICs.

Given the critical role of cognitive testing as the initial step in cognitive care, the potential of computerized assessments to increase test uptake, and the limited data on the integration of these tools in LMICs, we aimed to compare the validity of traditional paper-based and tablet-based cognitive assessment tools among aging Thai PWH. Additionally, we explored the acceptability and satisfaction of using tablet-based cognitive assessments, along with preferences for the different MoCA versions within this population.

## Methods

### Participants

PWH aged ≥ 50 years were enrolled from the HIV Netherlands Australia Thailand Research Collaboration (HIV-NAT), Thai Red Cross AIDS and Infectious Diseases Research Centre in Bangkok, Thailand, between April and May 2024. All participants were part of the HIV-NAT 006 study, an ongoing, prospective, clinic-based cohort that enrolled adults living with HIV (aged ≥ 18 years) established in 1996 (Clinicaltrials.gov NCT00411983, first registered on December 15, 2006) [13–17]. 

### Cognitive assessment and questionnaires

Cognitive performance was assessed using the Thai-validated versions of MoCA [8] and eMoCA [18]. The Thai paper-based MoCA is structurally similar to the original version, with content translated into Thai language. The eMoCA, administered via a tablet device, featured step-by-step animated instructions in Thai and contained content equivalent to the Thai paper-based MoCA. The adjusted cutoff score for detecting mild cognitive impairment was changed from < 26 to < 25, with an additional point awarded to participants with ≤ 6 years of education instead of ≤ 12 years as in the original MoCA. Additionally, a Thai-validated version of the Patient Health Questionnaire-9 (PHQ-9) [19] was used to assess depressive symptoms. Self-assessment questionnaires were developed for this study to assess participants’ touchscreen experience and their preference for the MoCA version (Supplementary File: Table S1 and Table S2).

### Study design

Participants were randomly assigned, using blocked randomization, in a 1:1 ratio to receive either the paper-based MoCA or the eMoCA during their first visit. Two weeks later, within an allowable window of ± 1 week, participants returned to the clinic to complete the alternate version of the MoCA. This interval was chosen to balance potential practice effects between the two test sequences [12]. The eMoCA was administered using an iOS-based tablet device. Additionally, all participants completed a set of self-assessment questionnaires regarding the touchscreen experience during the first visit. After completing the second visit, they were given questionnaires on acceptability, satisfaction, and preference.

### Standard protocol approvals

The study was approved by the institutional review board of the Faculty of Medicine, Chulalongkorn University, Bangkok, Thailand (IRB No. 781/66), and all participants or their proxies provided informed consent before any study procedures. All methods in this study were performed in accordance with the Declaration of Helsinki.

### Statistical analysis

Participants’ characteristics were summarized and categorized into two groups based on the MoCA version they completed first (paper-first and tablet-first groups). Pearson’s Chi-square test and the Mann-Whitney U test were used to compare characteristics between the two groups as appropriate. Multiple methods were used for validation analyses between the two MoCA versions. Discrepancy scores were calculated by subtracting the eMoCA score from the paper-based MoCA score for each participant. Pearson and partial correlation were used to determine the strength of the relationship between the paper-based MoCA and the eMoCA scores. Concordance correlation coefficients (CCC) were calculated, and a Bland-Altman plot was employed to determine the level of agreement between the two testing methods. Lastly, dependent t-tests were used to compare scores between modalities within groups and to compare scores between the first and second administrations within groups. All data analyses were performed using Stata/SE 17.0 (StataCorp LP, College Station, TX).

## Results

A total of 50 participants were enrolled in the study, with 4 participants excluded for not completing both tests. Consequently, 46 participants were included in the analysis: 24 in the paper-first group and 22 in the tablet-first group. The median age was 58.9 years (IQR: 55.0–62.0 years), 85% were male, and 15% had six years or less of education. The tablet-first group had a significantly higher proportion of males compared to the paper-first group (82% vs. 50%, *p* = 0.02) (Table 1). All participants reported current use of a touchscreen smartphone, but only 12 (26.1%) had experience using a touchscreen tablet. After completing both MoCA versions, 27 participants (60.0%) preferred the eMoCA, 11 (24.4%) preferred the paper-based MoCA, and 7 (15.6%) had no preference.

**Table 1:** Characteristics of all 46 participants, categorized by the version of the MoCA they first completed

Characteristics	Overall	Paper-first (*N* = 24)	Tablet-first (*N* = 22)	*p*
Age, years	58.9 (55.0–62.0)	59.3 (55.1–61.7)	58.7 (53.5–64.1)	0.96
Sex: Male	30 (65.2%)	12 (50.0%)	18 (81.8%)	0.02
Education ≤ 6 years	7 (15.2%)	6 (25.0%)	1 (4.5%)	0.05
Currently employed	38 (82.6%)	19 (79.2%)	19 (86.4%)	0.52
Income (Thai Baht per month)				0.92
< 10,000 (~ USD 300)	9 (19.6%)	5 (20.8%)	4 (18.2%)	
10,000–19,999 (~ USD 300–600)	11 (23.9%)	5 (20.8%)	4 (18.2%)	
≥ 20,000 (~ USD 600)	26 (56.5%)	14 (58.3%)	12 (54.5%)	
Marital status				0.02
Single	20 (43.5%)	7 (29.2%)	13 (59.1%)	
Married	16 (34.8%)	10 (41.7%)	6 (27.3%)	
Divorced/separated	4 (8.7%)	1 (4.2%)	3 (13.6%)	
Widowed	6 (13.0%)	6 (25.0%)	0 (0%)	
PHQ-9				0.75
No depression	26 (56.5%)	13 (54.2%)	13 (59.1%)	
Mild depression	14 (30.4%)	7 (29.2%)	7 (31.8%)	
Moderate depression	6 (13.0%)	4 (16.7%)	2 (9.1%)	
Hypertension	20 (44.4%)	10 (43.5%)	10 (45.5%)	0.89
Diabetes mellitus	21 (46.7%)	9 (39.1%)	12 (54.5%)	0.30
Dyslipidemia	39 (86.7%)	20 (87.0%)	19 (86.4%)	> 0.999
Chronic kidney disease	7 (15.9%)	4 (18.2%)	3 (13.6%)	> 0.999
HBV coinfection	4 (8.7%)	2 (8.3%)	2 (9.1%)	> 0.999
HCV coinfection	2 (4.3%)	2 (8.3%)	0 (0.0%)	0.49
Smoking status				> 0.999
Current smoker	4 (8.9%)	2 (8.7%)	2 (9.1%)	
Former smoker	11 (24.4%)	6 (26.1%)	5 (22.7%)	
Never	30 (66.7%)	15 (65.2%)	15 (68.2%)	
Alcohol consumption	3 (6.7%)	3 (13.0%)	0 (0%)	0.23
Nadir CD4 (cells/mm^3^)	197 (66–363)	197 (122–294)	228 (55–411)	0.76
Current CD4 (cells/mm^3^)	701 (472–881)	731 (472–915)	664 (465–860)	0.67
Duration of HIV infection (years)	24.8 (18.8–28.1)	26.8 (23.7–29.7)	24.0 (11.5–27.7)	0.07
Duration of ART (years)	23.4 (16.6–26.0)	23.8 (16.8–27.2)	23.3 (10.6–24.5)	0.36
HIV-1 RNA < 50 copies/mL	45 (100%)	23 (100%)	22 (100%)	> 0.999
Types of ART				
NRTI-based	44 (95.7%)	23 (95.8%)	21 (95.5%)	> 0.999
NNRTI-based	9 (19.6%)	8 (8.3%)	7 (31.8%)	0.07
INSTI-based	40 (87.0%)	23 (95.8%)	17 (77.3%)	0.09
Have ever used a touchscreen tablet	12 (26%)	5 (21%)	7 (32%)	0.40
Preference				
“I was frustrated when taking the paper and pencil version.”				0.26
Strongly disagree	5 (10.9%)	4 (16.7%)	1 (4.5%)	
Disagree	17 (37.0%)	6 (25.0%)	11 (50.0%)	
Neither agree nor disagree	20 (43.5%)	12 (50.0%)	8 (36.4%)	
Agree	3 (6.5%)	1 (4.2%)	2 (9.1%)	
Strongly agree	1 (2.2%)	1 (4.2%)	0 (0%)	
“I was frustrated when taking the computerized version.”				0.29
Strongly disagree	8 (17.4%)	6 (25.0%)	2 (9.1%)	
Disagree	20 (43.5%)	10 (41.7%)	10 (45.5%)	
Neither agree nor disagree	15 (32.6%)	7 (29.2%)	8 (36.4%)	
Agree	2 (4.3%)	0 (0%)	2 (9.1%)	
Strongly agree	1 (2.2%)	1 (4.2%)	0 (0%)	
The preferred version of the MoCA				0.07
Greatly prefer eMoCA	14 (31.1%)	11 (47.8%)	3 (13.6%)	
Prefer eMoCA	13 (28.9%)	5 (21.7%)	8 (36.4%)	
No preference	7 (15.6%)	2 (8.7%)	5 (22.7%)	
Prefer paper-based MoCA	7 (15.6%)	2 (8.7%)	5 (22.7%)	
Greatly prefer paper-based MoCA	4 (8.9%)	3 (13.0%)	1 (4.5%)	

Categorical data are presented as frequency (percentage) and continuous data as median (interquartile range)

Abbreviations: *ART* antiretroviral therapy, *HBV* hepatitis B virus, *HCV* hepatitis C virus, *INSTI* integrase strand transfer inhibitor, *MoCA* Montreal Cognitive Assessment, *NNRTI* non-nucleoside reverse transcriptase inhibitor, *PHQ-9* Patient Health Questionnaire-9, *USD* United States dollar

**Fig. 1:**
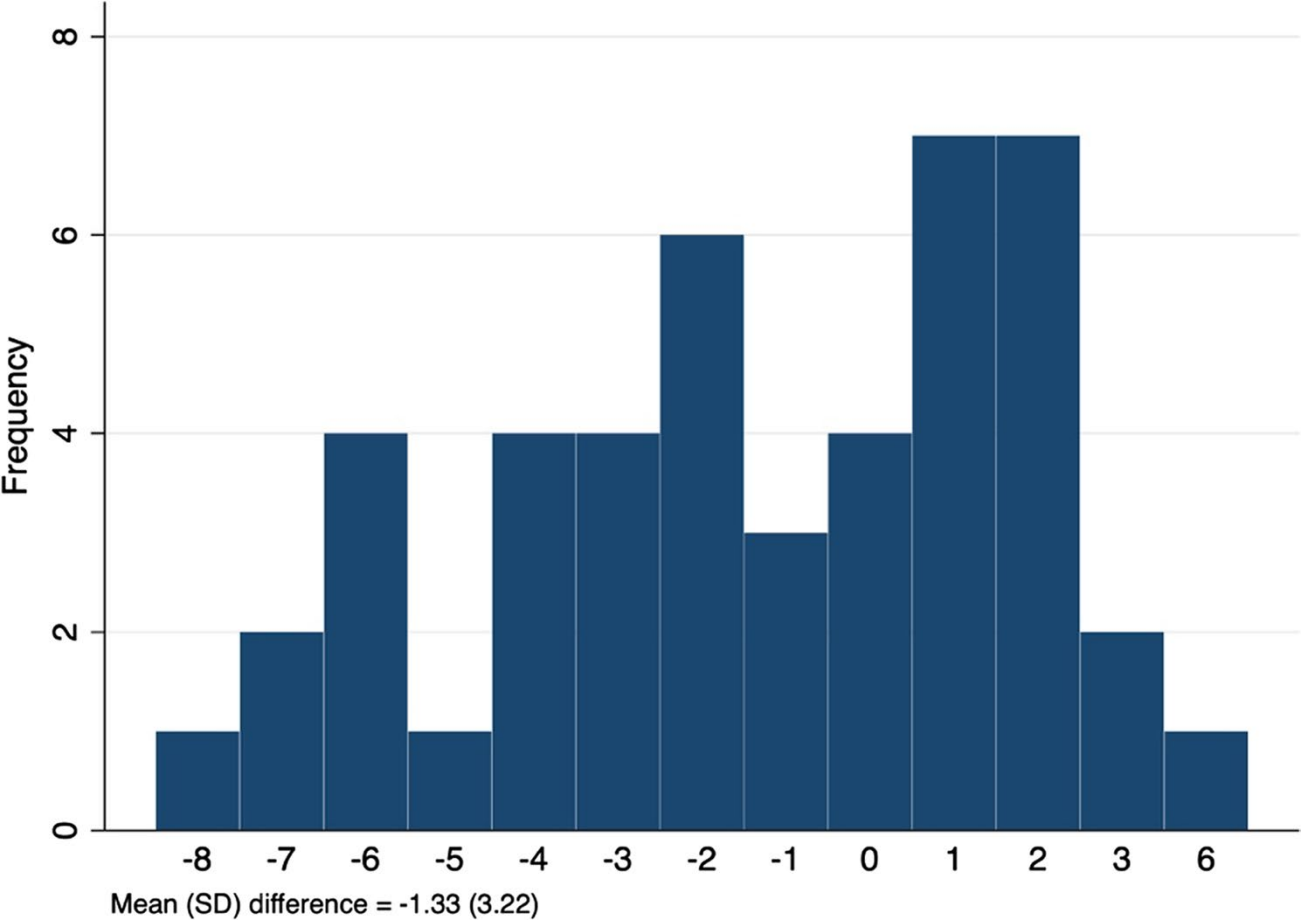
Distribution plot of differences between the total MoCA scores of each participant by paper-based MoCA and eMoCA

The mean (SD) total MoCA score from the paper-based MoCA was 25.30 (2.64), whereas the mean total MoCA score from the eMoCA was 23.98 (3.74). The discrepancy between the two MoCA versions of each individual ranged from − 8 to 6, with a mean difference of −1.33 (3.22). Approximately 30.4% had a difference of 1 point or less, and 58.7% had a difference within 2 points (Fig. 1). Both versions agreed on the classification of cognitive status, including cognitive impairment and cognitive unimpaired, in 63% of participants. However, 4.4% of participants were classified as having cognitive impairment only on the paper-based MoCA, while 32.6% were classified as having cognitive impairment only on the eMoCA.

**Fig. 2:**
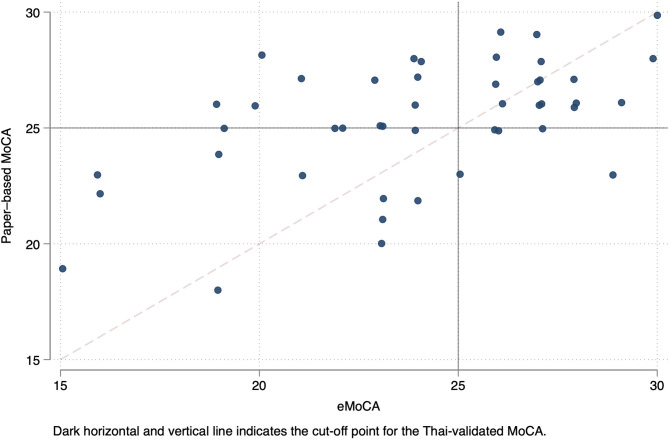
Correlation between each participant’s total MoCA score from paper-based MoCA and eMoCA

**Fig. 3:**
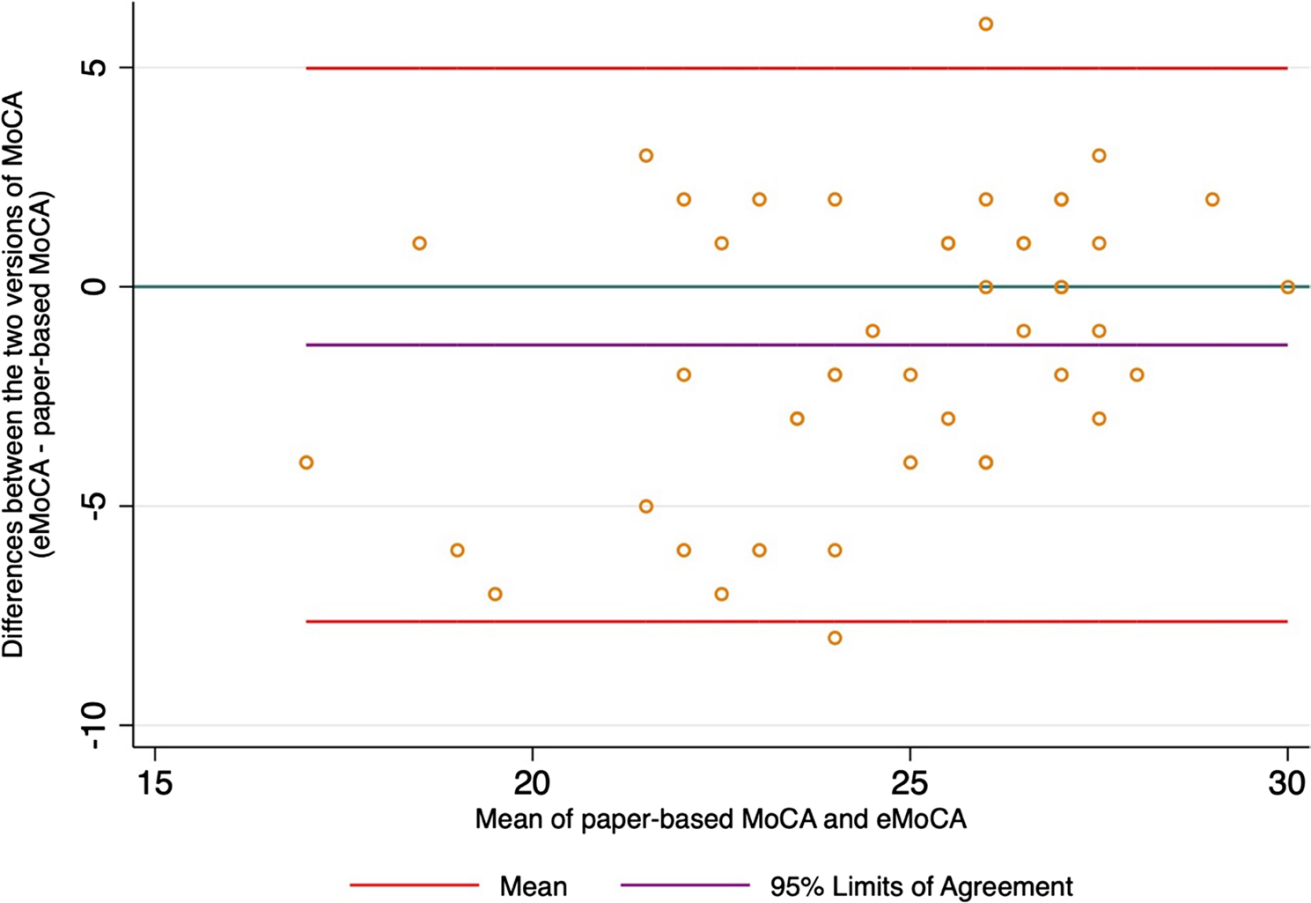
The Bland-Altman plot for data of the total MoCA scores of 46 participants, with the representation of the limits of agreement (red line), from − 1.96SD to + 1.96SD

Figure 2 demonstrates the total MoCA score from paper-based MoCA and eMoCA for all participants. Using Pearson correlation, there was a moderate correlation between participants’ total MoCA scores from the paper-based MoCA and the eMoCA (*r* = 0.54, *p* < 0.001). The strength of correlation increased among those who preferred the eMoCA over the paper-based MoCA (*r* = 0.62, *p* < 0.001), and no significant association was found among those who preferred the paper-based MoCA over the eMoCA (*r* = 0.45, *p* = 0.17). Among those who preferred the eMoCA, the strength of correlation was higher in participants with current employment (*r* = 0.70, *p* < 0.001). The CCC comparing total MoCA scores between the paper-based MoCA and the eMoCA was 0.47. The Bland-Altman plot showed 95% limits of agreement between − 7.63 and 4.98 **(**Fig. 3**)**. Furthermore, after adjusting for age, sex, and education, the partial correlation revealed a moderate relationship between the two MoCA versions (*r* = 0.47, *p* = 0.001).

Notably, the order of the MoCA administration influenced score changes between the two versions. In the paper-first group, there were no significant differences in total scores between the paper-based MoCA and the eMoCA (Supplementary File: Table S3). However, significant differences were observed in the domains of attention, delayed recall, and orientation. Conversely, the tablet-first group showed a significant improvement in total scores when switching from the eMoCA to the paper-based MoCA (from 23.73 to 26.78, mean difference 3.05, 95% CI, 1.86 to 4.23, *p* < 0.001), with significant differences in the domains of attention, abstraction, and delayed recall (Supplementary File: Table S4). There was a significant increase in delayed recall scores from the first to the second test in both groups, likely due to the learning effect of memorizing the same words across administrations.

Considering the differences between groups in the scores of the first test taken by each group (i.e., the paper-based MoCA for the paper-first group and the eMoCA for the tablet-first group), there were no significant differences in the mean total score or in any of the domain-specific scores throughout the MoCA (Supplementary File: Table S5).

Among participants with prior touchscreen tablet experience, total MoCA scores and all domain-specific scores between the paper-based MoCA and the eMoCA were comparable (total score: 25.75 vs. 25.08, mean difference − 0.67, 95% CI −2.53 to 1.20, *p* = 0.45), as shown in Table 2. Conversely, for those without touchscreen tablet experience, eMoCA scores were significantly lower than paper-based MoCA scores (25.15 vs. 23.59, mean difference − 1.56, 95% CI −2.72 to −0.40, *p* = 0.01). In this group, the attention domain was the only one with significant differences (5.50 vs. 4.79, mean difference − 0.71, 95% CI −1.10 to −0.31, *p* = 0.001) (Table 3).

**Table 2:** Comparison of total and domain-specific MoCA scores for participants who had used a touchscreen tablet before (*n* = 12)

	Paper-based MoCA	eMoCA	95% confidence interval of mean difference	*p*
Total score	25.75 (1.71)	25.08 (2.64)	−2.53 to 1.20	0.45
Visuospatial/executive	4.50 (0.67)	4.33 (0.98)	−0.76 to 0.43	0.55
Naming	3.00 (0)	3.00 (0)	0 to 0	–
Attention	5.75 (0.45)	5.67 (0.65)	−0.59 to 0.42	0.72
Language	1.58 (0.79)	1.58 (1.00)	−0.54 to 0.54	> 0.999
Abstraction	1.50 (0.52)	1.25 (0.45)	−0.64 to 0.14	0.19
Delayed recall	3.42 (0.79)	3.42 (1.44)	−1.01 to 1.01	> 0.999
Orientation	6 (0)	5.83 (0.39)	−0.41 to 0.08	0.17

**Table 3:** Comparison of total and domain-specific MoCA scores for participants who had never used a touchscreen tablet (*n* = 34)

	Paper-based MoCA	eMoCA	95% confidence interval of mean difference	*p*
Total score	25.15 (2.90)	23.59 (4.02)	−2.72 to −0.40	0.001
Visuospatial/executive	3.88 (1.23)	3.65 (1.25)	−0.60 to 0.13	0.20
Naming	2.97 (0.17)	2.88 (0.48)	−0.22 to 0.04	0.18
Attention	5.5 (0.66)	4.79 (1.32)	−1.10 to −0.31	0.001
Language	1.65 (1.12)	1.47 (1.11)	−0.47 to 0.11	0.23
Abstraction	1.32 (0.73)	1.21 (0.73)	−0.32 to 0.09	0.25
Delayed recall	3.62 (1.10)	3.41 (1.73)	−0.87 to 0.46	0.53
Orientation	6.00 (0)	5.91 (0.29)	−0.19 to 0.01	0.08

## Discussion

In validating the performance of the eMoCA compared to the traditional paper-based MoCA among aging Thai PWH in an HIV clinic, we found that the eMoCA demonstrated moderate correlation. The eMoCA appeared more challenging for the participants, with up to a third potentially diagnosed with mild cognitive impairment based solely on the eMoCA results. Importantly, scores between the two versions were comparable for participants with prior experience using a touchscreen tablet.

The moderate correlation between the two versions of the MoCA aligns with previous literature on individuals without HIV [12, 20] A prior study in people without HIV observed significant performance differences between groups during the second test, regardless of whether the paper or tablet version was administered first. In contrast, our study found significant differences between the two tests only in the tablet-first group. Notably, the larger increase in MoCA scores from the paper-based MoCA to the eMoCA, compared to the increase from the eMoCA to the paper-based MoCA, was consistent with previous findings. Besides the potential learning effect influencing both groups, we hypothesize that these significant differences may result from the more understandable and standardized instructions provided by the eMoCA, which utilized an instructional animation with slow speech and consistent wording, unlike the paper-based MoCA, in which delivery may vary depending on the test administrators.

Only one-fourth of the participants had prior experience using a touchscreen tablet, reflecting limited technological exposure among our aging population. In comparison, a study among aging people without HIV in the U.S. reported that 52.5% had prior tablet experience [11]. This may explain why a higher percentage of the participants were classified as having cognitive impairment using the eMoCA compared to the paper-based MoCA, illustrating the current considerations in implementing this method for patients in populations similar to ours. However, we found that those with prior touchscreen tablet experience had comparable scores between the two versions of the MoCA, particularly among those who were currently employed, possibly due to their recent exposure to touchscreen electronic devices in the workplace. With the expected increase in the number of aging individuals familiar with touchscreen tablets due to greater exposure during their younger years, it is optimistic that the eMoCA could achieve better performance in the future. Clinicians should also consider individuals’ level of touchscreen experience when selecting the administration modality.

Despite its moderate correlation, participants expressed high acceptability and satisfaction with the eMoCA, with more than half preferring it over the paper-based version. This is particularly noteworthy given that only 26% of participants had previous exposure to touchscreen tablets. For comparison, a previous study where half of the participants had prior tablet experience found that up to half reported no preference between the two MoCA versions [11]. This reflects our participants’ openness to integrating novel technologies into their care.

Certain limitations need to be considered in our study. First, while the MoCA is widely used, it may have limited sensitivity for detecting cognitive impairment specific to PWH, as it was not originally developed for this population. Second, the relatively small sample size warrants cautious interpretation of the results, and generalization of these findings to other populations should be approached carefully. Future studies with larger and more diverse participants, particularly regarding experience with the touchscreen tablets, are needed to validate and extend these findings.

## Conclusions

The eMoCA demonstrated moderate correlation compared to the paper-based MoCA, with prior experience using a touchscreen tablet significantly influencing the validity of the MoCA scores between the two versions, underscoring the importance of assessing touchscreen familiarity before selecting the test format. Despite the low number of participants with touchscreen tablet experience, the eMoCA was reported to be acceptable and satisfactory by participants. Given the expected increase in aging individuals familiar with touchscreen devices, there is optimism for improved performance of the eMoCA in the future. Therefore, while integrating the eMoCA into the model of care to expand diagnostic tool options, clinicians should also consider individuals’ level of touchscreen experience when choosing the appropriate MoCA administration method.

## Supplementary Information

Below is the link to the electronic supplementary material.

Supplementary Material 1

## Data Availability

The data that support the findings of this study are available from the corresponding author, AH, upon reasonable request.

## Abbreviations

AIDS: Acquired immunodeficiency syndrome
CCC: Concordance correlation coefficients
eMoCA: Electronic version of the MoCA
IQR: Interquartile range
HICs: High-income countries
HIV: Human immunodeficiency virus
HIV-NAT: HIV Netherlands Australia Thailand Research Collaboration
LMICs: Lower- to middle-income countries
MCI: Mild cognitive impairment
MoCA: Montreal Cognitive Assessment
PWH: People living with HIV
SD: Standard deviation
