# Supplementary material for: The validity of computerized Montreal cognitive assessment among aging people living with HIV: A pilot study
Source: BMC Neurol. 2025 Oct 1;25:406. doi: 10.1186/s12883-025-04425-9 (PMC12487254; doi:10.1186/s12883-025-04425-9)
Supplement: Supplementary file 1 — Supplementary Material 1 [file 12883_2025_4425_MOESM1_ESM.docx]

**Supplementary File**

**Table S1.** Self-assessment questionnaires for touchscreen experience.

| **EXPERIENCE WITH TOUCESCREEN DEVICES** (“Have the participant ever used _____ before?”) | | |
| --- | --- | --- |
| 5.1 Touchscreen phone | ○ Yes | ○ No (skipped to 5.2) |
| 5.1.1 Touchscreen phone usage  1 2 3 4 5 6 7 8 9 10  Rarely Very frequent | | |
| 5.2 Touchscreen tablet | ○ Yes | ○ No |
| 5.2.1 Touchscreen tablet usage  1 2 3 4 5 6 7 8 9 10  Rarely Very frequent | | |
| 5.2.2 Touchscreen tablet comfort  1 2 3 4 5 6 7 8 9 10  Not comfortable Very comfortable | | |

**Table S2.** Self-assessment questionnaires on MoCA version preference.

| **PREFERENCES QUESTIONNAIRE** |
| --- |

Using the following 1-10 scale, please answer items 1.1 – 1.5

1 2 3 4 5 6 7 8 9 10

*Strongly disagree* *Strongly agree*

|  | Items | Score |
| --- | --- | --- |
|  |  |  |
| 1.1 | I felt comfortable doing this assessment using the iPad. | _____ |
| 1.2 | The quality and clarity of the pictures was acceptable. | _____ |
| 1.3 | Being assessed using the iPad provides a true picture of how I am able to do things. | _____ |
| 1.4 | There were things I was unable to do/say because of the iPad that I was able to do/say using the pencil paper assessments. | _____ |
| 1.5 | If I had to have assessments or tests in the future, I would be willing to do them using an iPad. | _____ |

- 1. I was frustrated when taking the paper and pencil version.

| Strongly disagree | Disagree | Neither  agree nor disagree | Agree | Strongly agree |
| --- | --- | --- | --- | --- |


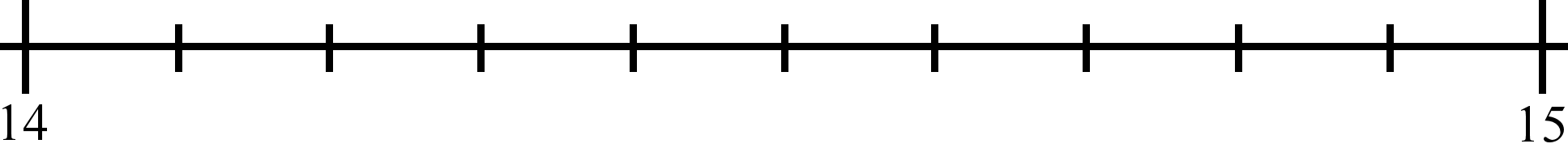


- 1. I was frustrated when taking the computerized version.

| Strongly disagree | Disagree | Neither  agree nor disagree | Agree | Strongly agree |
| --- | --- | --- | --- | --- |


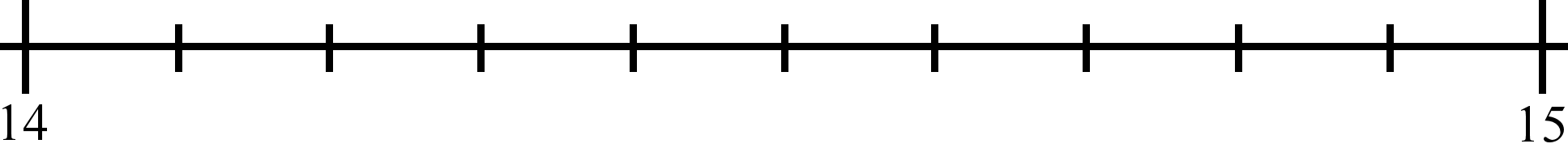


- 1. Which type of administration did you prefer?

○ Greatly prefer eMoCA

○ Slightly prefer eMoCA

○ No preference

○ Slightly prefer paper-based MoCA

○ Greatly prefer paper-based MoCA

**Table S3.** Comparison of total and domain-specific MoCA scores for the paper-first group (n = 24).

|  | **Paper-based MoCA** | **eMoCA** | **95% confidence interval of mean difference** | ***p*** |
| --- | --- | --- | --- | --- |
| Total score | 23.96 (2.69) | 24.21 (4.00) | -0.97 to 1.47 | 0.68 |
| Visuospatial/executive | 3.71 (1.30) | 3.67 (1.24) | -0.44 to 0.36 | 0.83 |
| Naming | 2.96 (0.20) | 2.83 (0.56) | -0.31 to 0.06 | 0.19 |
| Attention | 5.29 (0.69) | 4.75 (1.42) | -1.02 to -0.06 | 0.03 |
| Language | 1.42 (1.06) | 1.42 (1.14) | -0.37 to 0.37 | >0.999 |
| Abstraction | 1.13 (0.68) | 1.21 (0.66) | -0.13 to 0.30 | 0.43 |
| Delayed recall | 3.21 (0.88) | 4.21 (1.06) | 0.49 to 1.51 | <0.001 |
| Orientation | 6.00 (0) | 5.83 (0.38) | -0.33 to -0.01 | 0.04 |

**Table S4.** Comparison of total and domain-specific MoCA scores for the tablet-first group (n = 22).

|  | **eMoCA** | **Paper-based MoCA** | **95% confidence interval of mean difference** | ***p*** |
| --- | --- | --- | --- | --- |
| Total score | 23.73 (3.52) | 26.77 (1.63) | 1.86 to 4.23 | <0.001 |
| Visuospatial/executive | 4.00 (1.20) | 4.41 (0.80) | -0.06 to 0.88 | 0.08 |
| Naming | 3.00 (0) | 3.00 (0) | 0 to 0 | – |
| Attention | 5.32 (0.95) | 5.86 (0.35) | 0.08 to 1.01 | 0.02 |
| Language | 1.59 (1.01) | 1.86 (0.99) | -0.07 to 0.61 | 0.11 |
| Abstraction | 1.23 (0.69) | 1.64 (0.58) | 0.15 to 0.67 | 0.003 |
| Delayed recall | 2.55 (1.74) | 3.95 (1.05) | 0.74 to 2.07 | <0.001 |
| Orientation | 5.95 (0.21) | 6.00 (0) | -0.05 to 0.14 | 0.33 |

**Table S5.** Comparison of total and domain-specific MoCA scores from the first test taken between paper-first and tablet-first groups (n = 46).

|  | **Paper-first** | **Tablet-first** | **95% confidence interval of mean difference** | ***p*** |
| --- | --- | --- | --- | --- |
| Total score | 23.96 (2.69) | 23.73 (3.52) | -1.62 to 2.08 | 0.80 |
| Visuospatial/executive | 3.70 (1.30) | 4.00 (1.20) | -1.04 to 0.45 | 0.43 |
| Naming | 2.96 (0.20) | 3.00 (0) | -0.13 to 0.05 | 0.34 |
| Attention | 5.29 (0.69) | 5.32 (0.95) | -0.52 to 0.46 | 0.91 |
| Language | 1.42 (1.06) | 1.59 (1.01) | -0.79 to 0.44 | 0.57 |
| Abstraction | 1.13 (0.68) | 1.23 (0.69) | -0.51 to 0.30 | 0.61 |
| Delayed recall | 3.21 (0.88) | 2.54 (1.74) | -0.15 to 1.47 | 0.11 |
| Orientation | 6.00 (0) | 5.95 (0.21) | -0.04 to 0.13 | 0.30 |
